# The treatments and postoperative complications of esophageal cancer: a review

**DOI:** 10.1186/s13019-020-01202-2

**Published:** 2020-07-06

**Authors:** Qi-Liang Xu, Hua Li, Ye-Jing Zhu, Geng Xu

**Affiliations:** 1 Department of Cardiothoracic Surgery, Heze Municipal Hospital, Heze, 274031 Shandong China; 2 Department of Information, Heze Municipal Hospital, Heze, 274031 Shandong China; 3 Department of Clinical Pharmacy, Heze Municipal Hospital, Heze, 274031 Shandong China

**Keywords:** Esophageal cancer, Diagnosis, Risk factors, Postoperative complications, Anastomotic fistula

## Abstract

**Abstract:**

Esophageal cancer is still one of the most common cancers in the world. We review the appropriate treatments at different stages of esophageal cancer and also analyze the advantages and disadvantages of these treatments. The prognosis and recovery of different treatment regimens are further discussed. In particular, post-operative complications are the major causes of high mortality derived from the esophageal cancer. Therefore, we particularly discuss the main complications resulting in high mortality after surgery of esophageal cancer, and summarize their risk factors and treatment options.

**Background:**

As the common cancer, the complications of esophageal cancer after surgery have been not obtained systematic treatment strategy, focusing on treatment regimens based on the different stages of esophageal cancers.

**Methods and overview:**

This paper systematically summarizes the appropriate treatment strategies for different stages of esophageal cancers, and their advantages and disadvantages. We particularly focus on the postoperative survival rate of patients and postoperative complications, and discuss the causes of high mortality risk factors after surgery. The risk factors of death and corresponding treatment methods are further summarized in this study.

**Conclusion:**

Postoperative complications is the main cause responsible for the hard cure of esophageal cancers. The existing literatures indicate that postoperative anastomotic fistula is one of the most important complications leading to death, while it has not received much attention yet. We suggest that anastomotic fistula should be detected and dealt with early by summarizing these literatures. It is, therefore, necessary to develop a set of methods to predict or check anastomotic fistula in advance.

## Introduction

Esophageal cancer is one of the most common cancers worldwide and the sixth leading cause of cancer-related deaths [1, 2]. The incidence and mortality of esophageal cancers vary greatly from country to country, with significant regional differences [3]. In 2019, there were 328,030 new cases of digestive diseases in United States, with the death toll being expected to reach 165,460, among which there were only 17,650 new cases of esophageal cancer and 16,080 deaths [4]. It can be seen that esophageal cancer is a relatively rare disease in the western countries. As the most populous country over the world, the dataset of the incidence and mortality rate of cancers in China are very worth analyzing. According to 2018 statistics, 36.4% of digestive cancers, including stomach, liver and esophageal, in China have the very poor prognosis, and the 5-year overall survival rate is quite low (less than 35% from 2013 to 2015) [5]. By 2018, 283,433 people died of esophageal cancer in China, accounting for 9% of the total incidence of cancer. Although the incidence and mortality of esophageal cancer have been decreased in recent years [6], China is still one of the regions with high incidence of esophageal cancer over the world.

Esophageal carcinoma includes two major histological subtypes of esophageal squamous cell carcinoma (ESCC) and esophageal adenocarcinoma (EAC), and the majority of esophageal cancers belong to ESCC. Several factors are associated with the pathogenesis of esophageal cancer, with two most important factors of smoking and alcohol [7, 8]. In addition, poor diet, exposure to dangerous chemicals, and high-calorie beverages all enhance the risk of ESCC. Studies focusing on EAC have shown that Barrett’s esophagus (BE) lesions are a high risk factor resulting in EAC [9], and the risk of obesity has also increase the EAC by 2 to 3 times [10]. Therefore, early prevention of esophageal cancer is very meaningful, and it needs more social works to improve these conditions.

## Stages and treatment of esophageal cancer

Esophageal cancer has been proved to be one of the most difficult malignancies to treat. Although the improvement in surgery and decreases in perioperative mortality have been partially achieved during the therapy, meanwhile, the new model therapies have been pioneered and new technologies have been adopted [11, 12], these are still only beneficial in the early stage of prognosis [13]. In order to treat esophageal cancer, the treatment plan should be determined based on the stage of esophageal cancer. According to the 8th edition of the AJCC cancer staging manual, the stages of esophageal cancer are illustrated in Table 1. There are different treatment options for ESCC and EAC based on the diagnosis of esophageal cancer stages. Surgical treatment, radiotherapy and chemotherapy have been proved to effectively improve survival [14].

**Table 1:** Esophageal cancer/ Esophageal and gastroesophageal junction tumors. Application: squamous cell carcinoma, adenocarcinoma, adenosquamous carcinoma, undifferentiated carcinoma, neuroendocrine carcinoma, and adenocarcinoma with neuroendocrine differentiation. (excluding sarcomas, gastrointestinal stromal tumors)

**T--primary tumor**		**N--regional lymph nodes (for adenocarcinoma and squamous cell carcinoma)**	
T_X_	Primary tumors cannot be evaluated	N_X_	Regional lymph nodes cannot be evaluated
T_0_	No evidence of primary tumor	N_0_	No regional lymph node metastasis
T_is_	Highly atypical hyperplasia, confined to the epithelium	N_1_	One to two regional lymph node metastases
T_1_	The tumor invaded the lamina propria, mucous membrane or submucosa	N_2_	Three to six regional lymph node metastases
T_1a_	The tumor invades the lamina propria or the muscularis mucosa	N_3_	Equal to or more than 7 regional lymph node metastases
T_1b_	The tumor invaded the submucosa	**M--distal metastasis (for adenocarcinoma and squamous cell carcinoma)**	
T_2_	The tumor invaded the muscularis propria	M_0_	No remote transfer
T_3_	The tumor invaded the outer membrane	M_1_	There’s a distal shift
T_4_	The tumor invaded adjacent structures		
T_4a_	Tumor invades pleura, pericardium, azygos vein, transverse septum or pleura		
T_4B_	Tumors invade adjacent structures such as the aorta, vertebra, and trachea		
**G--degree of differentiation (adenocarcinoma, squamous cell carcinoma)**		**L--tumor location (tumor location refers to the center of the tumor, applicable to squamous cell carcinoma)**	
G_X_	The degree of differentiation cannot be assessed	X	unable to locate
G_1_	High differentiation	In the upper	Cervical esophagus to the lower margin of azygos vein
G_2_	moderately differentiated	In the middle	Inferior margin of azygos vein to inferior margin of pulmonary vein
G_3_	Low differentiation, undifferentiation	hypomere	Inferior pulmonary vein lower margin, to the gastroesophageal junction

Generally, the invasion degree of esophageal cancer is divided into different stages according to the diagnosis results, consequently, different decisions are provided for treatments. Endoscopic or surgical treatments are employed to BE lesions, endoscopic or surgical treatments are used for early cancer. And radiotherapy, chemotherapy or radio-chemoradiotherapy assisted surgery should be decided in the late stage of esophageal cancer according to the actual situation.

Surgical treatment is generally appropriate for the early cancer. Ablation methods are adopted for BE lesions, including laser therapy [15], photodynamic therapy (PDT) [12, 16], radiofrequency ablation [17], argon plasma ablation (APC) [18] and cryoablation [19]. Endoscopic mucosal resection (EMR) and endoscopic submucosal dissection (ESD) are the good options for lesions that do not penetrate the mucosal layer (T1a), or the lesions that develop only to the epithelium and lamina propria. While EMR is applicable to small tumors, and ESD is applicable to a wide range of lesions. Furthermore, endoscopic ultrasound (EUS) is employed to determine the degree of infiltration [20], which is more accurate than traditional ultrasound methods. Endoscopic therapy to save early esophageal cancer has been discussed in detail in previous studies [21–24], we will not elaborate here.

Esophagectomy is the most effective treatment for patients without invasion of adjacent organs or distant metastasis. According to the size and location of the tumor, the different surgical methods and treatment strategies can be selected. There are two kinds of esophagectomy, including open esophagectomy and minimally invasive esophagectomy. Three most common techniques for thoracic esophageal cancer include the transhiatal approach, Ivor Lewis esophagectomy (right thoracotomy and laparotomy), and McKeown technique (right thoracotomy followed by laparotomy and neck incision with cervical anastomosis) [25, 26]. It should be noted that some studies reported that the survival rate of patients with whole-piece esophagectomy is significantly higher than the patients with transhiatal esophagectomy. This means that the whole-piece esophagectomy is superior to transhiatal esophagectomy when the tumor is located in the lower esophagus or the cardia [27]. The surgical protocol for neck esophageal cancer is completely different from that for the chest. Neck esophageal cancer requires extensive lumpectomy, including hypopharynx, esophagus, larynx, thyroid gland, parathyroid gland, cervical lymph nodes and even permanent tracheotomy that is required in many cases, [28]. At present, the preferable treatment may be to reconstruct the esophagus by utilizing the portions of intestines or stomach [29, 30].

Minimally invasive esophagectomy includes various minimally invasive surgical methods aimed at reducing the trauma of esophageal surgery, represented by thoracoscopic / laparoscopic esophagectomy (TLE). At present, it also includes thoracoscopy/laparotomy, mediastinoscopy/laparoscopy, mediastinoscopy/laparotomy, and robot assisted minimally invasive surgery (RAMIE). With the continuous development of endoscopic equipment and technology, minimally invasive esophagectomy has been widely used. The results of TIME [31] and MIRO [32] show that, there was no significant difference in R0 resection rate, number of lymphadenectomy and 3 -, and 5-year survival rates between the patients treated by minimally invasive esophagectomy and open esophagectomy (P>0.05). The results showed that minimally invasive esophagectomy and open esophagectomy had the same effect on the degree of radical treatment of tumors. The minimally invasive esophagectomy can reduce the intraoperative blood loss, postoperative hospital stay and pain, and incidences of postoperative pulmonary infection and vocal coral paralysis. So it is worthy of clinical promotion and application.

The usage of chemotherapy as an auxiliary means can reduce the potential surgical staging and targeted micrometastasis of tumor for surgical treatment as well as the risk of metastasis. This type of chemotherapy is so called neoadjuvant chemotherapy, which is performed in the preoperative systemic chemotherapy, with the main purpose of shrinking the tumor and killing the metastatic cells, so as to ensure the smooth development of subsequent surgery. Moreover, Computed tomography (CT) and positron emission tomography (PET) are necessary to decide whether to use chemotherapy [33]. Recent PET studies suggested that low-radiation PET can complete 3D medical imaging of the whole body within only 30 s [34], which further demonstrates the superiority of this technology. In addition, some patients still have local recurrence or distant metastasis after surgery, thus, a lot of efforts have been made to use adjuvant chemotherapy to reduce recurrence or metastasis and improve prognosis. Adjuvant chemotherapy (AC) that puts potentially small metastases in the body under the control of chemotherapy drugs earlier may be more beneficial to their elimination. According to a study based on 3592 patients with esophageal carcinoma (84.7% adenocarcinoma, 15.2% squamous cell carcinoma), from which 335 (9.3%) were treated with esophagectomy AC, the result shows that AC was not related to a significantly reduced risk of death in patients without residual disease or residual non-lymph node disease. In the rest of lymphadenopathy patients, the risk of death derived from AC and the entire cohort was reduced by 30%. Moreover, some patients with postoperative length of stay ≤10 days and no unexpected readmission, the risk of death in AC and residual lymphadenopathy was reduced by about 40% [35]. Another study also showed that postoperative AC contributes to the prognosis of patients with stage II or III esophageal cancer [36].

Concurrent chemoradiotherapy is the most standard treatment for esophageal cancer patients who cannot be treated by surgical resection. After reviewing 2667 references, two randomized studies were identified by six reports. Based on available evidence, combined esophagectomy with chemoradiotherapy for locally advanced esophageal squamous cell carcinoma has little or no difference in overall survival, which is possibly due to higher treatment-related mortality [37]. Recently, there is obvious evidences demonstrate that locally advanced esophageal cancer could lead to gastric mucosal injury and bleeding after radiotherapy and chemotherapy [38]. Therefore, only patients who need radiotherapy and chemotherapy for clear surgical stage can be chosen by this treatment plan.

## Prognosis and recovery of esophageal carcinoma

Prognosis is an old topic. Most patients with esophageal cancer have advanced to the middle or late stage when they seek medical treatment. The 5-year survival rate after treatment for early esophageal cancer is 90%, while the 5-year survival rate of patients for middle or late stage is only 6% ~ 15%. The rarity of cancer (< 1% of cancers) and the significant differences in histological cell types and esophageal location between the eastern and western institutions have hindered the study of the treatment of esophageal cancer. Thus, the selection of appropriate treatment on the basis of improving the detection rate of early lesions is the key stage to reduce the disease burden and improve the prognosis of patients. Here we systematically review the prognostic effects of different current treatments for esophageal cancers.

### The prognosis of surgical treatment

Radical surgical treatment is an ideal treatment for early esophageal cancer, and the postoperative survival rate is more than 90%. In 2009, the global collaboration on esophageal cancer (WEOC) published the results of 7884 esophagectomy cases of esophageal cancer from 13 participating institutions, in which 4725 have not received AC nor radiotherapy. After 30 days, 1 year, 5 years and 10 years, the overall survival rates were 98, 78, 42 and 31%, respectively [39]. The death risk is higher in the first 2 years after esophagectomy, and then declines at a constant rate of 5.9% per year [40]. Saddoughi et al. [40] conducted a retrospective analysis of 3500 cases of esophagectomy from 1985 to 2013, and found that 52 cases (1.5%) of patients with stage IV esophageal cancer received surgical resection, with the 1-year and 5-year survival rate of 29 and 6%, respectively. Thus, esophagectomy should not be performed for stage IV disease, and the palliative treatment may be more suitable.

### Endoscopic therapy

#### Endoscopic resection

EMR is a method to extract large masses of mucosal tissue after injecting normal saline or epinephrine into the submucosa of the lesion to form a liquid pad. It is principally applicable to the resection of flat and polypoid early gastrointestinal tumors, and has dual diagnostic and therapeutic effects. EMR is also the most widely used endoscopic resection at present because of its small trauma, simple operation and few complications. Ciocirla et al. [41] retrospectively analyzed 34 patients with T1 stage esophageal cancer who received repeated EMR treatment, and the results showed that 31 patients were complete remission. While other 3 patients showed dilatation stenosis, no distal metastasis and local recurrence, with the 5-year survival rate being 95%. However, there are still some limitations of the treatment of early esophageal cancer by utilizing EMR. For example, due to the limitation of the size of resectable tissues under endoscope, block resection should be adopted if the diameter of the lesion is greater than 20 mm, but it is easy to exert residual tumor cells on the edge of the resected tissue [42].

ESD is a new technology developed on the basis of EMR. Its major method is to use high-frequency electrotome, such as TF knife, Dual knife and Hook knife, to peel off the pathological mucosa according to endoscopic mucosal injection, with aim to achieve the goal of radical treatment of tumor. In comparison with EMR, ESD can not only completely remove a wide range of lesions (diameter greater than 20 mm), but also provide complete pathological diagnosis data. ESD has become the main treatment method for early esophageal cancer and precancerous lesions during recent years. Probst et al. [43] investigated 111 cases of early esophageal cancer treated by ESD, including 87 cases of adenocarcinoma and 24 cases of squamous cell carcinoma, with an average follow-up time of 24.3 months and 38.0 months, respectively. The survival rate of adenocarcinoma and squamous cell carcinoma patients was 97.766 and 95.866%, respectively, and the overall survival rate was 96.666 and 66.766%, respectively. In the process of postoperative follow-up, 2 patients presented esophageal stenosis and bleeding. Toyonaga et al. [44] conducted follow-up analysis on 1635 patients receiving ESD treatment (including 111 cases of early esophageal cancer and precancerous lesions), and found that no recurrence or serious complications occurred during the median follow-up time, with the 5-year survival rate of the patients being 81.6%.

#### Nonexcision therapy

RFA is the radiofrequency wave of medium and high frequency emitted by the radiofrequency tip inserted into the diseased tissue, which causes shock, friction and heat production of tissue cell ions, and also results in denaturation, necrosis and coagulation of tumor tissue and cells rapidly under high temperature, thereby achieving the purpose of treating tumor. Van Vilsteren et al. [45] treated 13 patients with early esophageal cancer and precancerous lesions using RFA, and demonstrated that 3 patients with minor postoperative complications did not need treatment again. All the patients had no recurrence during the follow-up period and had a normal life, with a median follow-up time of 17 months.

PDT is a method of irradiating tumor tissue containing a photosensitizer with a specific wavelength of laser light after intravenous injection of the photosensitizer. The tissue oxygen then reacts to form highly reactive singlet oxygen and free radicals. Through cytotoxic effects, many biological macromolecules in tumor cells are destroyed, leading to tumor cell apoptosis and necrosis. Prasad et al. [46] retrospectively examined the treatments of 199 cases of precancerous esophageal lesions, including 70 cases of esophagectomy and 129 cases of PDT. The results indicated that the mortality rate was 9 and 8.5% for the PDT group and surgery group, respectively, suggesting insignificant discrepancies. It can be seen that the long-term efficacy of PDT is comparable to that of esophagectomy patients because of the small trauma and fewer complications. Therefore, the minimally invasive treatment method is worthy of promotion for early esophageal cancer.

APC is a non-contact electric coagulation technology. Argon gas is ionized into good conductivity of argon ion in the context of the action of high frequency voltage, ionized argon ion beam electrode of the high frequency current flows to the target tissue to generate high frequency electric coagulation effects. Then the dispersion characteristics of argon ion beam can be automatically limited to excessive solidification on a larger scale to achieve the effects of uniform solidification. Madisch et al. [47] followed up 70 patients with early esophageal cancer treated by APC, with the follow-up time being 102 months. Among70 patients, 7 patients relapsed and 6 patients received additional treatment, and the 5-year overall survival rate was 81.6%. These results indicate that APC is safe and effective for the treatment of early esophageal squamous cell carcinoma, especially in elderly patients and patients with surgical contraindications.

### Adjuvant therapy and neoadjuvant therapy

Surgical treatment is utilized in the treatment of esophageal cancer at the present time,while the simple surgical treatment is not effective and the survival rate is also low. Meanwhile, most patients are already in the middle and late stages of the disease when they are diagnosed, with being often accompanied by local or different parts of the body metastasis. Since the symptoms of early esophageal cancer patients are insidious and difficult to be found. Hence, the simple surgery is difficult to obtain sufficient clinical effect. Neoadjuvant therapy prior to esophagectomy is the standard treatment for locally advanced and operable esophageal cancer. Neoadjuvant chemotherapy, which is also known as initial chemotherapy, refers to the reduction of tumor size through chemotherapy before surgery, this can reduce tumor load and control distant metastasis as early as possible. Existing studies have shown that preoperative neoadjuvant chemotherapy can significantly improve the short-term and long-term survival rate of patients [48, 49]. Fluorouracil and paclitaxel combined with platinum and platinum, respectively, are two commonly used chemotherapy regimens for esophageal cancer [49]. Stiles et al. [50] reviewed the survival status of 238 patients who received neoadjuvant therapy or chemotherapy and radiotherapy before resection during 1987–2013. Results showed that 15% (*n* = 36) died within 1 year after esophagectomy, and 69% died in documented recurrence of cancer. Saddoughi et al. [40] analyzed the causes of esophagectomy after neoadjuvant therapy and believed that the performance state, poor tumor differentiation and lack of response to neoadjuvant therapy were risk factors leading to early death. Tong et al. [51] performed multivariate scores on 175 patients who received surgery after neoadjuvant chemoradiotherapy, and suggested a high percentage of remaining active tumor cells in the primary tumor, moreover, the positive lymph node was an independent predictor of poor prognosis. Neoadjuvant chemoradiotherapy has the characteristics of double-edged sword. Sufficient responders may have better survival in the long term, while the diseases may occur in the process of neoadjuvant therapy for the patients with adverse reactions. It is, therefore, meaningful to determine the clinical, biochemical and molecular predictors of tumor regression after neoadjuvant therapy [49].

## Postoperative complications

Esophageal cancer is serious not only because it is difficult to cure, but also it is easy to be accompanied by a variety of complications. The existence of various complications of esophageal cancer makes esophageal cancer an extremely serious disease that is difficult to cure, and the harm to patients is continuous. At present, Surgery is the preferred treatment for patients with esophageal cancer. However, the influences of surgery and postoperative treatments are prone to induce complications of esophageal cancer. In this section, we primarily discuss functional gastric emptying disorder, severe diarrhea and reflux esophagitis, pulmonary infection, chylothorax, and anastomotic fistula as well as other complications.

### Functional gastric emptying disorder (FDGE)

In general, some patients with esophageal cancer also need to remove the gastric wall, or even a part of the stomach, when removing the esophagus. Since the esophagus is connected with the stomach, with the mutual influences on the function. The resection of esophageal cancer is prone to gastric movement disorders, thereby leading to obstacle of empty of function of bosom stomach and bringing about content of a large number of stomach to detain. It is reported that more than 50% of patients after esophagectomy have FDGE symptoms [52]. Different anastomotic modes are correlated with the occurrence of FDGE [53]. Gender differences, longer operation time and hospital stay are also have the probability of becoming factors contributing to FDGE [54]. In addition, a study of 285 patients undergoing esophagectomy suggests that the incidence of FDGE was 18.2%, and gastric size (gastric tube versus the whole stomach) was the only significant factor influencing the incidence of FDGE among perioperative factors [55]. The previous research showed that gastric tube was significantly better than the whole stomach in reducing the incidence of postoperative gastric emptying disorder (PDGE) [56].

### Reflux esophagitis

Reflux esophagitis is a common postoperative complication of esophageal cancer, it is mainly manifested as body flexion after the meal or acid liquid and food reflux from the stomach and esophagus to the pharynx or mouth when patients sleep in bed at night. This is also accompanied by symptoms such as post-sternal burning, pain, difficulty in swallowing and so on. The occurrence of this symptom may be related to vagotomy and gastrin concentration [57]. Besides, anastomosis level is correlated with reflux esophagitis. In the investigation of 53 patients who have thoracic esophageal cancer and underwent root canal esophagectomy, gastric tube reconstruction and neck anastomosis, results showed that anastomosis degree of gastric tube reconstruction after esophageal cancer was associated with the incidence of reflux esophagitis [58].

### Pulmonary complication

Although the lung tissue was not resected in the surgery for esophageal cancer, the integrity of the thoracic wall and the intercostal muscles are damaged, especially for the integrity of the diaphragm, since the ventilation pump of the affected lung was severely damaged, respiratory tract infection is easy to occur. Patients may experience varying degrees of dyspnea and shortness of breath after surgery when pain from neck, chest, or upper abdominal incisions occurs, if the stomach is pulled into the chest to compress the lungs. The pathophysiological mechanisms lead to pulmonary infection including alveolar collapse, pulmonary edema, weakened pulmonary defense mechanisms, and poor ventilation [59]. Perioperative risk factors associated with postoperative pulmonary infection include chronic bronchitis, chronic cardiac insufficiency, and age ≥ 80 years [60].

The recent studies demonstrated that 28% of 2704 patients occurred severe respiratory complications, 15% appeared pneumonia, and 7% occurred respiratory failure [61]. Elliott et al. [62] analyzed the impacts of neoadjuvant therapy on postoperative pulmonary function, and found that neoadjuvant therapy altered pulmonary physiology, especially for the diffusion ability. These may lead to pulmonary complications, and also show a potentially modifiable risk index, which also demonstrates that neoadjuvant therapy is a potential risk factor for complications. Most of patients with esophageal cancer lung infection have postoperative pulmonary edema, which increase sputum production, ineffective cough and sputum cough. Assisting patients to effectively sputum cough is one of the main measures to treat pulmonary infection. Patients should be encouraged to drink water and give intravenous fluids to increase body hydration. However, for the patients with certain organic heart diseases, such as coronary heart disease and hypertension, infusion rates should be reduced based on the changes in heart rate and blood pressure in daily observation. Pain caused by chest wall incision and drainage tube can make patients worry about pain, reduce the depth of breathing, lead to rejection of cough or weak cough, and damage to the ipsilateral pulmonary ventilation function, which prevent effective release and relief of sputum cough. Meanwhile, the application of effective antibiotic therapy is undoubtedly one of the most effective methods to control pulmonary infection [63].

### Chylothorax

Postoperative chylothorax is still an important factor for reoperation and prolonged hospitalization after esophageal cancer. Chylothorax is potentially life-threatening and difficult to treat. It is an uncontrolled chylothorax caused by tissue damage to the thoracic duct during the operation process of esophageal cancer, and most often occurs between 2 and 10 days after surgery. Chylous drainage from catheter injury can lead to wound infection, skin flap necrosis, chylothorax, and other severe cases, it also can result in carotid artery exposure and rupture [64]. When this happens, closed thoracic drainage should be installed, and drainage volume should be closely observed, meanwhile, medium-chain triglyceride diet or total parenteral nutrition (TPN) nutrition improvement treatment should be given to maintain hydrolytic balance and supplement nutrition. In these conditions some patients may cure after treatment [65]. For the patients with large chylous flow, the chylous duct should be ligated immediately after thoracotomy [66]. Lin et al. [67] provided a method of intraoperative selective thoracic ligation of thoracic duct, which could reduce the incidence of postoperative chylothorax. Some studies reported that low fat-containing elemental formula is effective for postoperative recovery and potentially useful to prevent chyle leak [68]. Several cases take early intervention to reduce mortality after esophagectomy by conducting minimally invasive surgery [69].

### Anastomotic or thoracogastric fistula

Anastomotic fistula is a serious postoperative complication of esophageal cancer, with an incidence of 8.2–15% [70–72]. The reasons are derived from the anastomotic mode, tension of the anastomotic site, secondary infection of the anastomotic site, and nutritional status of the patient before the operation. Neck anastomotic fistula is not easy to threaten the patient’s life, and can be healed by drainage. Intrathoracic anastomotic fistulas often occur 5–10 days after surgery. They can even lead to pleural membrane pollution and gastric necrosis when the condition worsens, causing a great threat to patients and a high mortality rate, even though some anastomotic fistulas are asymptomatic.

The surgical strategy of esophagectomy has been gradually improved, nevertheless, some potential risk factors still lead to postoperative anastomotic fistula. Anastomotic stoma or gastric stump fistula after esophageal cancer, leading to local inflammatory cell infiltration, poor mucosal healing, and further develop into fistula, mostly due to infiltration and infiltration of inflammatory substances in the suture plane, local erosion of digestive juice or bleeding in the suture plane. This is a process that gradually progresses from occult to fistula. According to the case analysis of this group, the fistula is mostly local inflammation or small rupture in the early stage, which is “occult”. The common reason is that the pressure of the stapling device or the closure device is uneven, resulting in local fracture and damage of the anastomotic mucosa layer. Another cause is that a hematoma is formed when the stump of the local gastric wall is closed, leading to poor blood flow in the suture plane, and poor local healing of the anastomosis or gastric stump. Because the fistula is small and there is only a small amount of digestive juice or gas in the gastrointestinal tract, patients often only have persistent sepsis such as elevated body temperature, increased heart rate, and fatigue. The location of the anastomotic site has a certain impact on the occurrence of anastomotic fistula. Neck anastomosis has a higher leakage rate (25–45%) compared with intrathoracic anastomosis (5–15%), which may lead to an increase in recurrent nerve palsy and longer hospital stay [73, 74]. Female patients with postoperative hypoproteinemia and renal insufficiency are more likely to have anastomotic fistula [75].

Note that the optimal treatment for anastomotic leakage is unclear. Previous studies argued that conservative treatment, oral and intravenous antibiotics, drainage, and surgical and non-surgical treatment can be used according to the patient’s situation and treatment unit preference [76, 77]. Surgical repair can be tried and reinforced with greater omentum or intercostal muscle flap when the patients are in early anastomotic fistula, it should be noted that the mortality rate of the second operation is higher [78]. If the amount of leakage is small, endoscopic surgery can be performed [79], such as Endoscopic vacuum-assisted closure (EVAC) [80]. EVAC is based on the continuous negative pressure applied to the wound by sponge [81], so that the anastomotic site can be anastomosed again or the leakage site can be closed to achieve the purpose of treatment, which has the advantages of effectively attracting influenza dye and accelerating wound healing. In addition, a retrospective study of 70 patients, who have esophageal anastomotic leakage after Ivor Lewis esophagectomy treated by self-expanding metal stents (SEMS), found that the treatment of this method with SEMS was effective, safe and technically feasible [82]. At present, the application of SEMS in the treatment of anastomotic fistula after resection of esophageal cancer has become another mature treatment method [82–84]. Embedding anastomosis with epiploon is an effective way to reduce the anastomotic fistula after esophagectomy, and it will not cause additional damage to cardiopulmonary function.

Prevention of anastomotic leakage should focus on preoperative nutritional status and intraoperative operations to minimize direct catheter trauma for the postoperative management. The longer time for gastric tube reconstruction are more likely to suffer from ischemia at its tip, as esophageal reconstruction mainly involves the stomach. It is therefore necessary to pay attention to the preoperative severing of gastric vessels. Furthermore, improving perioperative management and early postoperative enteral nutrition, pulmonary physiotherapy, prevention of hypoxemia and hypotension are all the important measures to reduce the incidence of anastomotic leakage [76]. Patient status should be closely monitored after surgery, and the patients with fever or leukocytosis should be vigilant. Atrial fibrillation may be a strong indicator of leakage, and the high levels of inflammatory markers in the blood are also possible indicators of leakage. Once the anastomotic fistula occurs, it should be diagnosed as soon as possible. Generally, the selected methods include esophagography, upper gastrointestinal endoscopic examination, computed tomography (CT), etc. In the early stage of postoperative microfistula, inflammatory reactants temporarily “closed” the fistula, and the gastric circumference became smaller after surgery. Especially for the tubular stomach, the contrast agent often passes through the digestive tract quickly, most of the contrast agent has no obvious extravasation, and it is easy to miss the diagnosis of intrathoracic fistula. If the stomach contents are not fully decompressed or given food, it is easy to enlarge the fistula and progress into a severe fistula. While the spiral CT examination is non-invasive and does not stimulate the wound margin, which can clearly show the hierarchical structure around the digestive tract in the chest. In recent years, particularly in thin-layer CT, scattered small gas or a small amount of uneven density of effusion around the anastomotic or gastric incision edge appear, and the presence of hidden thoracic fistula is often considered in the clinical manifestations of systemic poisoning. A prospective clinical study confirms that CT shows that small gas shadows around the thoracic and digestive tract are significantly superior in the sensitivity of the diagnosis of occult fistula in the chest, compared to the leakage of contrast agent in upper gastrointestinal angiography. And a set of CT-based predictive score has been proposed by some doctors to provide higher accuracy for the diagnosis of anastomotic fistula [85].

### Anastomotic stenosis

Anastomotic stenosis is a common complication after radical resection of esophageal cancer. Although postoperative anastomotic stenosis does not immediately endanger the life of patients, it seriously affects the quality of life of patients. Most of the anastomotic stenosis is caused by scar contraction, as results of too much anastomotic suture, the tight suture, and the not well matched mucosa. Anastomotic leakage, multi-layer anastomosis and long-term fluid diet are the high risk factors that can cause anastomotic stenosis after operation of esophageal cancer. Attention should be paid to avoid the occurrence of these factors before, during and after operation, which can effectively reduce the occurrence of anastomotic stenosis. Expanding narrow anastomotic stoma is a common method at early clinical stage. At this time, the scar is not firm and easy to expand, but expansion is prohibited within 1 month to prevent anastomotic leakage [86].

### Severe diarrhea

Esophageal cancer surgery may also lead to severe diarrhea by causing gastrointestinal dysfunction. Antidiarrheal drugs should be given actively, along with rehydration to prevent dehydration.

## Conclusions

Esophageal cancer is one of the most common malignant tumors of the digestive tract. There are differences in regions, races and pathological types worldwide. Generally, according to the results of diagnosis, the invasion degree of esophageal cancer is divided into stages, and different treatment decisions are provided accordingly. In the early stage of cancer, endoscopic or surgical treatment is used, while in the late stage, radiotherapy, chemotherapy or adjuvant surgery should be decided according to the actual situation.

There are two methods of esophageal cancer operation: open resection and minimally invasive resection. Early esophageal cancer also has certain mortality, since the postoperative complications is difficult to cure. Complications can directly affect the postoperative efficacy and quality of life of patients. Complications of esophageal cancer surgery mainly include chylothorax, pulmonary complications, anastomotic leakage, anastomotic stenosis and reflux esophagitis. Some patients with esophageal cancer may have functional gastric emptying disorder. We think that gastroplasty is a better way to prevent functional gastric emptying disorder after esophageal cancer operation. The incidence of postoperative pulmonary complications (PPC) of esophageal cancer is high, and the harm is serious. Patients’ age, obesity, smoking and lung diseases can lead to the occurrence of pulmonary complications. Preoperative respiratory function training, reduction of lung injury during operation and application of postoperative drugs can reduce the incidence of pulmonary complications. Chylothorax after esophageal cancer surgery is rare. But once it happens, the consequences of chylothorax are very serious, because of chylous fluid leakage and the lost of a large number of body fluids. It is safe and reliable to apply the low-set and supradiaphragmatic en bloc ligation of surrounding tissues with the thoracic duct for prevention of chylothorax following esophagectomy. Meanwhile, anastomotic fistula is a kind of postoperative complications that must be vigilant. The appearance of fistula is a gradual process after the operation of esophageal and cardia carcinoma, from occult to typical thorax fistula. Therefore, we need to pay attention to thoracic occult fistula, occult fistula is found as early as possible and standard conservative treatment is performed through examinations of the chest CT scan and gastrointestinal angiography. These can significantly reduce the incidence of thoracic fistula after the surgery of esophageal cancer and cardia cancer, and reduce the mortality of patients with anastomotic fistula.

Anastomotic stenosis is a common complication after radical resection of esophageal cancer. If there are problems in anastomotic suture during surgical treatment, anastomotic leakage and long-term fluid diet after operation can cause anastomotic stenosis. Therefore, it is of great clinical significance for the prevention of anastomotic stenosis to adopt a reasonable operation and guide a reasonable diet after esophagectomy. There are important measures to reduce the incidence of postoperative complications and mortality by strengthen the management of respiratory tract, aseptic operation and gastric tissue protection, postoperative enteral and enteral nutrition support, and early out of bed activities.

## Data Availability

Please contact author for data requests.

## Abbreviations

ESCC: esophageal squamous cell carcinoma
AC: adjuvant chemotherapy
EUS: endoscopic ultrasound
FDGE: functional gastric emptying disorder
SEMS: self-expanding metal stents
EVAC: endoscopic vacuum-assisted closure

## References

[1] Bray F (2018). Global cancer statistics 2018: GLOBOCAN estimates of incidence and mortality worldwide for 36 cancers in 185 countries. CA Cancer J Clin.

[2] Jemal A (2011). Global cancer statistics. CA Cancer J Clin.

[3] Ferlay J (2015). Cancer incidence and mortality worldwide: sources, methods and major patterns in GLOBOCAN 2012. Int J Cancer.

[4] Siegel RL, Miller KD, Jemal A (2019). Cancer statistics, 2019. CA Cancer J Clin.

[5] Zeng H (2018). Changing cancer survival in China during 2003-15: a pooled analysis of 17 population-based cancer registries. Lancet Glob Health.

[6] Chen W (2016). Cancer statistics in China, 2015. CA Cancer J Clin.

[7] Feng RM (2019). Current cancer situation in China: good or bad news from the 2018 global Cancer statistics?. Cancer Commun (Lond).

[8] Enzinger PC, Mayer RJ (2003). Esophageal cancer. N Engl J Med.

[9] Ronkainen J (2005). Prevalence of Barrett's esophagus in the general population: an endoscopic study. Gastroenterology.

[10] Cook MB (2010). Cigarette smoking and adenocarcinomas of the esophagus and esophagogastric junction: a pooled analysis from the international BEACON consortium. J Natl Cancer Inst.

[11] Kato H (2007). Surgical treatment for esophageal cancer. Current Issues Dig Surg.

[12] Agostinis P (2011). Photodynamic therapy of cancer: an update. CA Cancer J Clin.

[13] Ono S (2009). Long-term outcomes of endoscopic submucosal dissection for superficial esophageal squamous cell neoplasms. Gastrointest Endosc.

[14] Rustgi AK, El-Serag HB (2014). Esophageal carcinoma. N Engl J Med.

[15] Smith MS, et al. Volumetric laser endomicroscopy and its application to Barrett's esophagus: results from a 1,000 patient registry. Dis Esophagus. 2019;32(9):1–8. 10.1093/dote/doz029 PMC6853704 31037293

[16] Wu H, Minamide T, Yano T. Role of photodynamic therapy in the treatment of esophageal cancer. Dig Endosc. 2019;31(5):508–16. 10.1111/den.13353 30667112

[17] Pohl H (2009). Endoscopic versus surgical therapy for early cancer in Barrett's esophagus: a decision analysis. Gastrointest Endosc.

[18] Pech O (2017). Hybrid argon plasma coagulation in patients with Barrett esophagus. Gastroenterol Hepatol (N Y).

[19] Thota PN (2018). Cryotherapy and radiofrequency ablation for eradication of Barrett's esophagus with dysplasia or Intramucosal Cancer. Dig Dis Sci.

[20] Ajani JA (2011). Esophageal and esophagogastric junction cancers. J Natl Compr Cancer Netw.

[21] Noordzij IC, Curvers WL, Schoon EJ (2019). Endoscopic resection for early esophageal carcinoma. J Thorac Dis.

[22] Rizvi QU (2017). Endoscopic Management of Early Esophagogastric Cancer. Surg Oncol Clin N Am.

[23] Naveed M, Kubiliun N (2018). Endoscopic treatment of early-stage esophageal Cancer. Curr Oncol Rep.

[24] Smith I, Kahaleh M (2015). Endoscopic versus surgical therapy for Barrett's esophagus neoplasia. Expert Rev Gastroenterol Hepatol.

[25] Wright CD (2005). Esophageal cancer surgery in 2005. Minerva Chir.

[26] Rentz J (2003). Transthoracic versus transhiatal esophagectomy: a prospective study of 945 patients. J Thorac Cardiovasc Surg.

[27] Hagen JA, Peters JH, DeMeester TR (1993). Superiority of extended en bloc esophagogastrectomy for carcinoma of the lower esophagus and cardia. J Thorac Cardiovasc Surg.

[28] Peracchia A (2001). Current status of surgery for carcinoma of the hypopharynx and cervical esophagus. Dis Esophagus.

[29] Miyata H (2013). Larynx-preserving limited resection and free jejunal graft for carcinoma of the cervical esophagus. World J Surg.

[30] Lee HS (2012). Free jejunal graft for esophageal reconstruction using end-to-side vascular anastomosis and extended pharyngo-jejunostomy. Ann Thorac Surg.

[31] Mariette C, Markar SR, Dabakuyo-Yonli TS (2019). Hybrid minimally invasive esophagectomy for esophageal cancer. N Engl J Med.

[32] Straatman J, van der Wielen N, Cuesta MA (2017). Minimally invasive versus open esophageal resection:three-year follow-up of the previously reported randomized controlled trial:the TIME trial. Ann Surg.

[33] Kato H (2005). The incremental effect of positron emission tomography on diagnostic accuracy in the initial staging of esophageal carcinoma. Cancer.

[34] Reardon S (2019). Whole-body PET scanner produces 3D images in seconds. Nature.

[35] Burt BM (2017). Utility of adjuvant chemotherapy after Neoadjuvant Chemoradiation and Esophagectomy for esophageal Cancer. Ann Surg.

[36] Sohda M (2019). Post-esophagectomy adjuvant chemotherapy benefits esophageal Cancer patients. In Vivo.

[37] Vellayappan BA (2017). Chemoradiotherapy versus chemoradiotherapy plus surgery for esophageal cancer. Cochrane Database Syst Rev.

[38] Monma S, et al. Gastric mucosal injury and hemorrhage after definitive chemoradiotherapy for locally advanced esophageal cancer. Esophagus. 2019;16(31):402–7. 10.1007/s10388-019-00680-1 31222680

[39] Rice TW (2009). Worldwide esophageal cancer collaboration. Dis Esophagus.

[40] Saddoughi SA (2017). Survival after surgical resection of stage IV esophageal Cancer. Ann Thorac Surg.

[41] Ciocirlan M (2007). Endoscopic mucosal resection for squamous premalignant and early malignant lesions of the esophagus. Endoscopy.

[42] Ishihara R (2008). Comparison of EMR and endoscopic submucosal dissection for en bloc resection of early esophageal cancers in Japan. Gastrointest Endosc.

[43] Probst A (2015). Early esophageal cancer in Europe: endoscopic treatment by endoscopic submucosal dissection. Endoscopy.

[44] Toyonaga T (2013). 1,635 endoscopic submucosal dissection cases in the esophagus, stomach, and colorectum: complication rates and long-term outcomes. Surg Endosc.

[45] van Vilsteren FG (2011). Radiofrequency ablation for the endoscopic eradication of esophageal squamous high grade intraepithelial neoplasia and mucosal squamous cell carcinoma. Endoscopy.

[46] Prasad GA (2007). Long-term survival following endoscopic and surgical treatment of high-grade dysplasia in Barrett's esophagus. Gastroenterology.

[47] Madisch A (2005). Long-term follow-up after complete ablation of Barrett's esophagus with argon plasma coagulation. World J Gastroenterol.

[48] Ma S (2018). Neoadjuvant chemotherapy followed by minimally invasive esophagectomy is safe and feasible for treatment of esophageal squamous cell carcinoma. Thorac Cancer.

[49] Wong C, Law S (2017). Predictive factors in the evaluation of treatment response to neoadjuvant chemoradiotherapy in patients with advanced esophageal squamous cell cancer. J Thorac Dis.

[50] Stiles BM (2015). Clinical predictors of early cancer-related mortality following neoadjuvant therapy and oesophagectomy. Eur J Cardiothorac Surg.

[51] Tong DK (2010). Histological regression of squamous esophageal carcinoma assessed by percentage of residual viable cells after neoadjuvant chemoradiation is an important prognostic factor. Ann Surg Oncol.

[52] Poghosyan T (2011). Functional disorders and quality of life after esophagectomy and gastric tube reconstruction for cancer. J Visc Surg.

[53] Sutcliffe RP (2008). Anastomotic strictures and delayed gastric emptying after esophagectomy: incidence, risk factors and management. Dis Esophagus.

[54] Benedix F (2017). Risk factors for delayed gastric emptying after esophagectomy. Langenbeck's Arch Surg.

[55] Zhang L (2017). Risk factors for delayed gastric emptying in patients undergoing esophagectomy without pyloric drainage. J Surg Res.

[56] Crestanello JA, Deschamps C, Stephen D (2005). Selective management of intrathoracie anastomotic leak after esophageetomy. J Thorac Cardiovasc Surg.

[57] Rostas JW (2011). T.T. Mai, and W.O. Richards, *Gastric motility physiology and surgical intervention*. Surg Clin North Am.

[58] Sakai M (2017). Impact of the level of anastomosis on reflux esophagitis following Esophagectomy with gastric tube reconstruction. World J Surg.

[59] Lunardi AC (2012). Weakness of expiratory muscles and pulmonary complications in malnourished patients undergoing upper abdominal surgery. Respirology.

[60] Grotenhuis BA (2010). Preoperative risk assessment and prevention of complications in patients with esophageal cancer. J Surg Oncol.

[61] Low DE (2019). Benchmarking complications associated with Esophagectomy. Ann Surg.

[62] Elliott JA, et al. Effect of neoadjuvant chemoradiation on preoperative pulmonary physiology, postoperative respiratory complications and quality of life in patients with oesophageal cancer. Br J Surg. 2019;106(10). 10.1002/bjs.11218 31282584

[63] Yu Z (2018). Society for Translational Medicine Expert Consensus on the prevention and treatment of postoperative pulmonary infection in esophageal cancer patients. J Thorac Dis.

[64] Ilczyszyn A, Ridha H, Durrani AJ (2011). Management of chyle leak post neck dissection: a case report and literature review. J Plast Reconstr Aesthet Surg.

[65] de Gier HH (1996). Systematic approach to the treatment of chylous leakage after neck dissection. Head Neck.

[66] Lagarde SM (2005). Incidence and management of chyle leakage after esophagectomy. Ann Thorac Surg.

[67] Lin Y (2017). Selective en masse ligation of the thoracic duct to prevent Chyle leak after Esophagectomy. Ann Thorac Surg.

[68] Moro K (2016). Low fat-containing elemental formula is effective for postoperative recovery and potentially useful for preventing chyle leak during postoperative early enteral nutrition after esophagectomy. Clin Nutr.

[69] Hayden JD (2007). Minimally invasive management of chylous fistula after esophagectomy. Dis Esophagus.

[70] Zhou C (2015). Superiority of minimally invasive Oesophagectomy in reducing in-hospital mortality of patients with Resectable Oesophageal Cancer: a meta-analysis. PLoS One.

[71] van Workum F (2017). Improved functional results after minimally invasive Esophagectomy: Intrathoracic versus cervical anastomosis. Ann Thorac Surg.

[72] Singhal S (2017). Simple technique of circular stapled anastomosis in Ivor Lewis Esophagectomy. J Laparoendosc Adv Surg Tech A.

[73] Siewert JR, Stein HJ, Bartels H (2004). Anastomotic leaks in the upper gastrointestinal tract. Chirurg.

[74] Blewett CJ (2001). Anastomotic leaks after esophagectomy for esophageal cancer: a comparison of thoracic and cervical anastomoses. Ann Thorac Cardiovasc Surg.

[75] Huang J (2017). Logistic regression analysis of the risk factors of anastomotic fistula after radical resection of esophageal-cardiac cancer. Thorac Cancer.

[76] Chen KN (2014). Managing complications I: leaks, strictures, emptying, reflux, chylothorax. J Thorac Dis.

[77] Messager M (2017). Recent improvements in the management of esophageal anastomotic leak after surgery for cancer. Eur J Surg Oncol.

[78] Fahn HJ (1997). Leakage of intrathoracic oesophagovisceral anastomoses in adenocarcinoma of the gastric cardia: changes in serial APACHE II scores and their prognostic significance. Eur J Surg.

[79] Schubert D (2006). Endoscopic treatment of mediastinal anastomotic leaks. Zentralbl Chir.

[80] Min YW (2019). Endoscopic vacuum therapy for postoperative esophageal leak. BMC Surg.

[81] Wedemeyer J (2008). Endoscopic vacuum-assisted closure of upper intestinal anastomotic leaks. Gastrointest Endosc.

[82] Plum PS (2019). Outcome of self-expanding metal stents in the treatment of anastomotic leaks after Ivor Lewis Esophagectomy. World J Surg.

[83] Eizaguirre E (2016). Treatment of anastomotic leaks with metallic stent after esophagectomies. Dis Esophagus.

[84] Fernandez A (2015). Fully covered metal stents for the treatment of leaks after gastric and esophageal surgery. Rev Esp Enferm Dig.

[85] Goense L (2017). Diagnostic performance of a CT-based scoring system for diagnosis of anastomotic leakage after esophagectomy: comparison with subjective CT assessment. Eur Radiol.

[86] Jun L, Jibiao H (2003). Experiences in prevention and management of anastomotic stenosis during esophageal cancer surgery. J Practical Med.

